# Comparison of indirect markers of insulin resistance in adult patients with Double Diabetes

**DOI:** 10.1186/s12902-020-00570-z

**Published:** 2020-06-15

**Authors:** Aldo Ferreira-Hermosillo, Raúl Ibarra-Salce, Joshua Rodríguez-Malacara, Mario Antonio Molina-Ayala

**Affiliations:** 1 Unidad de Investigación Médica en Enfermedades Endocrinas, Hospital de Especialidades Centro Médico Nacional Siglo XXI, Instituto Mexicano del Seguro Social (IMSS), Cuauhtémoc No. 330, Colonia Doctores, Mexico City, Mexico; 2 Servicio de Endocrinología, Hospital de Especialidades Centro Médico Nacional Siglo XXI, Instituto Mexicano del Seguro Social (IMSS), Cuauhtémoc No. 330, Colonia Doctores, Mexico City, Mexico

**Keywords:** Diabetes mellitus, type 1, Metabolic syndrome, Insulin resistance

## Abstract

**Background:**

The presence of insulin resistance (IR) and metabolic syndrome (MS) in patients with type 1 diabetes (T1D) has been called “double diabetes”. This entity increases the risk for development of micro and macrovascular complications and cardiovascular mortality. The gold standard for IR quantification is the hyperinsulinemic euglycemic clamp (HEC) but it is invasive, time-consuming and not available in the majority of the clinical settings. Because of this, some formulas for IR quantification have been proposed. We aimed to compare the utility of those methods for MS detection in patients with T1D.

**Methods:**

We conducted a cross-sectional study in 112 patients with T1D and determined the presence of MS using the Joint Statement Criteria. We calculated the estimated glucose disposal rate (eGDR), estimated insulin sensitivity index (eIS), natural logarithm of glucose disposal rate (lnGDR), triglyceride/high-density lipoprotein cholesterol ratio (TG/HDL-c), visceral adipose index (VAI) and waist-to-height ratio (WHtR), and compared among patients with and without MS using Student t-test or Mann-Whitney U test. Receiver Operating Characteristics curves for the different indexes were used to identify the best cut-off points for MS detection.

**Results:**

Thirty three percent of the patients were considered to have MS. The patients with MS had lower eGDR (5.49 [4.37–6.80] vs. 8.93 [8.03–9.94] mg/kg/min), eIS (2.89 [1.54–3.54] vs. 3.51 [2.68–4.68]) and lnGDR (1.69 ± 0.27 vs. 1.95 ± 0.21 mg/kg/min), and higher WHtR (0.55 ± 0.05 vs. 0.50 ± 0.05), VAI (3.4 [1.92–5.70] vs. 1.39 [0.97–1.92]) and TG/HDL-c (3.78 [2.63–5.73] vs. 1.77 [1.18–2.75]) in comparison with patients without MS. The cut-off points of TG-HDL-c > 2.0, eGDR < 7.32 mg/kg/min, lnGDR < 1.8 mg/kg/min, VAI > 1.84, WHtR > 0.52 and eIS < 2.92 had a sensitivity of 86, 85, 82, 77 and 70% respectively, for MS detection. The TG/HDL-c, lnGDR and eIS sensitivity changed depending on sex meanwhile eGDR, WHtR and VAI did not need adjust by sex.

**Conclusion:**

Our data show that an eGDR < 7.32 mg/kg/min have the highest sensitivity and specificity to detect the presence of MS in patients with T1D.

## Background

Type 1 diabetes (T1D) is an autoimmune disease characterized by pancreatic β-cell destruction, resulting in absolute insulin deficiency. On the other hand, type 2 diabetes (T2D) is associated with varying degrees of insulin resistance (IR) and relative insulin deficiency [1]. Recently, some studies have reported patients with T1D that develop clinical features of T2D as obesity, hypertension, dyslipidemia, or metabolic syndrome (MS) [2]. This phenomena is called “double diabetes” and has been associated with an increased rate of chronic complications and cardiovascular diseases in patients with T1D [3, 4].

The gold standard to measure insulin resistance is the glucose disposal rate (GDR) derived from the hyperinsulinemic euglycemic clamp (HEC) [5, 6]. However, clamps are invasive and time-consuming procedures, which are mainly performed for research purposes. In patients with T2D, indirect methods as the homeostasis model assessment of insulin resistance (HOMA-IR) and the Matsuda index have demonstrated to be useful for the assessment of IR [7], but their usage is limited in patients with T1D because both require a preserved β-cell function. Other formulas have been proposed for the assessment of IR in patients with T1D.

The first validated and most used method is the estimated glucose disposal rate (eGDR) [8], in which lower rates correlate with greater IR [9]. Furthermore, in patients with T1D it predicts the development of chronic complications as nephropathy, peripheral vascular disease and coronary artery disease [10]. In the Mexican population, our group found that an eGDR below 7.32 mg/kg/min had a 80% sensitivity for MS diagnosis [11]. We also demonstrated that the waist-to-height ratio (WHtR, with a cut-off point of > 0.52) is helpful to predict the presence of MS [12]. Other available methods are the insulin sensitivity prediction equation (eIS) [13]; the natural logarithm of the GDR [14]; the triglycerides/high density lipoprotein cholesterol (TG/HDL-c) ratio and the visceral adiposity index (VAI) [15]. All of these indexes are simple to calculate, they are based on widely available clinical parameters, and they have been validated in different populations, showing adequate sensitivities and specificities when compared to the HEC. Nevertheless, no studies have compared their utility for the detection of MS. The purpose of this study was to evaluate and compare the utility of those indexes for the detection of MS in patients with T1D and to propose specific cut-off values for this population.

## Methods

We performed a cross-sectional evaluation in all the patients of the Type 1 Diabetes Clinic from January 2018 to January 2019 (Hospital de Especialidades Centro Médico Nacional Siglo XXI, a tertiary care referral center). We included patients that were 18 years of age or older at the time of the study, with at least 3 visits per year to the clinic and no change in insulin dose during the same time. Patients with incomplete records or follow-up, poor treatment adherence and primary dyslipidemias were excluded. We also excluded patients in treatment with metformin, thiazolidinediones or SGLT-2 (sodium glucose co-transporter type 2) inhibitors. Data such as age at diagnosis, family history, tobacco or alcohol use, quantity of consumed kilocalories, minutes of exercise performed, and type and doses of insulin where directly interrogated to each participant. The presence of microvascular complications (nephropathy, neuropathy and retinopathy), and gout was obtained from their clinical records. The study completed all the requirements by the local ethics committee and was conducted in accordance with the Declaration of Helsinki regarding Ethical Principles for Medical Research Involving Humans. All the patients gave their written consent.

### Diagnostic criteria for MS

Patients were considered to have MS when they presented 3 or more of the Joint Statement Criteria [16]: hypertriglyceridemia (triglycerides [TG] > 150 mg/dl [1.7 mmol/l] or in treatment for this condition); low serum high-density lipoprotein cholesterol (HDL-c < 40 mg/dl [1.03 mmol/l] in men or < 50 mg/dl [1.29 mmol/l] in women]; hypertension (blood pressure higher than 130/85 mmHg or in treatment for this condition); and central obesity (defined using WC), as previously described [11]. All the patients met the criteria of high fasting glucose since they had T1D.

### Anthropometric measurements

A single investigator registered the weight (kg), height (meters), and WC (cm) and calculated the WHtR, waist-to-hip ratio (WHR) and body mass index (BMI). BMI was used to classify weight groups, according to the World Health Organization (WHO) criteria [17]. Blood pressure was determined as the average value after two measurements with a 5-min difference between them.

### Biochemical determinations

We analyzed glucose, total cholesterol (TC), HDL-c and TG with a commercially available kit using photocolorimetry. Glycated hemoglobin (HbA1c) was evaluated by turbidimetric immunoanalysis [11]. Low-density lipoprotein cholesterol (LDL-c) was calculated with Friedewald formula: LDL-c (mg/dl) = TC mg/dl – (HDL-c mg/dl + TG mg/dl/5) if TG were < 400 mg/dl [18]. Serum creatinine was evaluated with a commercial kit and glomerular filtration rate (GFR) was estimated using CKD-EPI equation [19].

### Insulin resistance quantification methods

Insulin resistance was calculated using the following methods:

- Estimated glucose disposal rate (eGDR): 24.31 – (12.22 x waist-to-hip ratio [WHR]) – (3.29 x hypertension [defined as 0 = no, 1 = yes]) – (0.57 x HbA1c [%]). Using this formula, a lower eGDR level indicates greater IR [9].
- Estimated insulin sensitivity (eIS): exp. 4.1075–0.1299 (waist, cm) – 1.05819 (daily insulin dose, U/kg) – 0.00354 (TG, mg/dl) – 0.00802 (DBP, mmHg). Lower values indicate decreased insulin sensitivity [13].
- Natural logarithm of glucose disposal rate (lnGDR) = 4.964–0.121 x HbA1c (%) – 0.012 x diastolic blood pressure, DBP (mmHg) – 1.409 x WHR. A low lnGDR indicates greater IR [14].
- TG/HDL-c ratio was calculated from the lipid profile by dividing TG by HDL-c using values in units of mg/dl. Higher ratios suggest IR [15].
- Visceral adiposity index (VAI) was calculated for women = [WC/36.58 + (1.89 * BMI)] * (TG in mg/dl/0.81) * (1.52/HDL-c in mg/dl) and for men = [WC/39.68 + (1.88*BMI)] * (TG in mg/dl/1.03) * (1.31/HDL-c in mg/dl). Higher VAI values indicate IR [15].

### Sample size calculation

We calculated the sample size using the comparison of proportions formula, considering that 37% of our patients with T1D had metabolic syndrome as previously reported [12]. For this purpose, we considered a confidence level of 95%, margin of error of 5% and a limited population size of 150 patients (representing all the patients of our Clinic). The sample size required was 106 patients.

### Statistical analysis

Data were analyzed using SPSS v. 23. We evaluated normality using a Kolmogorov-Smirnov test. Results are expressed as mean ± standard deviation (SD) or medians (interquartile ranges). Associations between quantitative variables were assessed with Student t test or Mann-Whitney U test. Qualitative variables (as the frequency of hypertension, hypertriglyceridemia, low HDL-c levels, obesity, gout, hypercholesterolemia, microvascular diseases, and KDIGO [Kidney Disease: Improving Global Outcomes] stages in patients with or without MS) were associated with chi-squared or Fisher’s test. Receiver Operating Characteristic (ROC) curves were used to identify the best cutoff point of each IR index using Youden formula (sensitivity + specificity − 1) [20]. We also evaluated the area under the ROC curve (AUC, with 95% confidence intervals). A *p* < 0.05 was considered to be significant.

## Results

We evaluated 112 patients during the study period. The median age of our population was 35 years (26–43 years), 66% were females and the median time since diagnosis was 22 years (15–29 years). Regarding their family history, 75% had first-degree relatives with T2D, 64% with hypertension and 10% with dyslipidemia. Twenty two percent of the patients smoke more than one cigarette per day and 27% reported regular alcohol consumption. In the whole group 33% had hypertension, 14% hypertriglyceridemia, 31% had low HDL-c levels (according sex) and 21% had hypercholesterolemia (defined as TC levels higher than 200 mg/dl). Using the WHO classification, 31% of the patients had overweight or obesity, and according to the IDF proposed cut-off points, 36% had central obesity [median WC in men of 87 cm (81–97 cm) and in women of 81 cm (75–88 cm)]. The patients reported a previous assessment by a nutritionist with a diet adherence of 70% (43–80%). Only 18% practiced regular exercise (more than 150 min per week). All the patients were exclusively treated with insulin, with an intensive regimen: 60% used long-acting insulin analogue (glargine 100 U/ml), 36% used neutral protamine Hagedorn insulin (NPH) and 2% were on treatment with mixed insulin. Regarding prandial insulin, 83% used lispro and 16% used regular insulin. The median dose of insulin was 43 U/day (32–60 U/day) or 0.70 U/kg/day (0.54–0.96 U/kg/day).

According the Joint Statement Criteria, 33% of the patients (*n* = 37) were considered to have MS. Table 1 compares the baseline characteristics of the groups with and without MS. We observed that patients with MS were older, had higher weight, BMI, waist and hip circumference (with concomitant higher WHR), had higher concentrations of triglycerides and higher systolic and diastolic blood pressure in comparison with the patients without MS. We also observed that patients with MS had lower HDL-c concentration, but this difference was only significant in females. When comparing patients with and without MS, there were no significant differences in family history of T2D (74% vs. 75%), hypertension (71 vs. 58%) or dyslipidemias (12 vs. 1%), prevalence of hypothyroidism (49% vs. 36%), and type and doses of insulin (72% vs. 54% of patients with vs. without MS were on treatment with glargine, 64% vs. 73% were on treatment with lispro, respectively). There were no differences in glucose or HbA1c levels among groups. As expected, the prevalence of all the components of MS, including gout, were different among groups. Sixty percent of patients with MS in comparison with 32% of patients without MS had overweight or obesity. Despite there were no differences among groups in the prevalence of previous diagnosis of microvascular complications, the glomerular filtration rate (GFR) was lower and the KDOQI stages were worst in patients with MS.

**Table 1:** Baseline characteristics of the population comparing patients with and without MS

	Total population (*n* = 112)	Without MS (*n* = 75)	With MS (*n* = 37)	*p*^a^
Female sex	66 (74)	64 (48)	70 (26)	NS
Age, years	35 (26–43)	34 (25–40)	37 (31–47)	0.035
Time since diagnosis, years	22 (15–29)	22 (14–28)	24 (16–38)	NS
Insulin dose:				
U/day	43 (32–60)	42 (31–54)	52 (34–65)	NS
U/kg/day	0.70 (0.54–0.96)	0.69 (0.53–0.96)	0.72 (0.53–0.93)	NS
Weight, kg	63 (55–71)	60 (54–69)	66 (58–78)	0.007
Height, m	1.62 ± 0.89	1.62 ± 0.92	1.62 ± 0.85	NS
BMI, kg/m^2^	24.2 ± 3.3	23.4 ± 2.7	25.9 ± 3.8	< 0.001
WC, cm	84 (78–91)	79 (74–87)	88 (83–97)	< 0.001
Female	81 (75–88)	78 (74–85)	85 (81–92)	0.002
Male	87 (81–97)	83 (78–87)	97 (90–102)	0.001
Hip circumference, cm	98 (93–103)	97 (92–101)	100 (94–105)	0.014
WHR	0.86 ± 0.08	0.84 ± 0.06	0.89 ± 0.07	< 0.001
Female	0.83 ± 0.06	0.82 ± 0.06	0.86 ± 0.05	0.028
Male	0.90 ± 0.07	0.87 ± 0.05	0.96 ± 0.06	< 0.001
SBP, mmHg	106 ± 15	104 ± 14	110 ± 17	0.027
DBP, mmHg	71 ± 9	69 ± 8	74 ± 10	0.017
FBG, mg/dl	133 (80–222)	138 (79–232)	126 (85–195)	NS
HbA1c, %	8.61 ± 1.73	8.4 ± 1.6	9.0 ± 1.8	NS
TG, mg/dl	113 (78–172)	94 (70–135)	172 (123–249)	< 0.001
TC, mg/dl	169 (142–195)	169 (142–190)	165 (141–204)	NS
HDL-c, mg/dl	51 (44–63)	56 (48–68)	45 (37–49)	< 0.001
Female	57 (45–67)	61 (51–72)	45 (38–53)	< 0.001
Male	49 (40–54)	49 (40–56)	47 (35–49)	NS
LDL-c, mg/dl	89 (73–111)	88 (73–110)	92 (75–111)	NS
GFR, ml/min/1.73 m^2^SC	74.7 (43.7–106.8)	77.6 (57.6–116.6)	52.6 (28.9–91.1)	0.029
Metabolic Syndrome and Comorbidities, %(n)				
Hypertension	33 (37)	16 (12)	68 (25)	< 0.001
Hypertriglyceridemia	14 (16)	0	43 (16)	< 0.001
Low HDL-c	31 (35)	16 (12)	62 (23)	< 0.001
Central obesity	36 (41)	23 (17)	65 (24)	< 0.001
Female	26 (30)	17 (13)	46 (17)	< 0.001
Male	10 (11)	5 (4)	19 (7)	0.020
Obesity (BMI)				0.029
Normal	65 (72)	74 (55)	46 (17)	
Overweight	24 (27)	19 (14)	35 (13)	
Class I Obesity	7 (7)	4 (3)	14 (5)	
Class II Obesity	1 (1)	0	3 (1)	
Hypercholesterolemia	21 (24)	13 (10)	38 (14)	0.006
Gout	4 (5)	0	14 (5)	0.003
Microvascular disease				
Nephropathy	14 (16)	12 (9)	19 (7)	NS
Neuropathy	13 (14)	9 (7)	19 (7)	NS
Retinopathy	7 (8)	5 (4)	11 (4)	NS
KDIGO Stages:				0.044
Stage 1	47 (53)	51 (38)	41 (15)	
Stage 2	25 (28)	29 (22)	16 (6)	
Stage 3	17 (19)	15 (11)	22 (8)	
Stage 4	6 (7)	4 (3)	11 (4)	
Stage 5	4 (5)	1 (1)	11 (4)	

Data are presented as %(n), mean ± SD or median (IQR). *Statistically different among patients with or without MS with Student t test, Mann Whitney U, chi squared or Fisher’s test, depending on their type and distribution. *MS* metabolic syndrome, *BMI* body mass index, *WC* waist circumference, *SBP* systolic blood pressure, *DBP* diastolic blood pressure, *FBG* fasting blood glucose, *TG* triglycerides, *TC* total cholesterol, *HDL-c* high density lipoprotein cholesterol, *LDL-c* low density lipoprotein cholesterol, *GFR* glomerular filtration rate, *KDIGO* Kidney Disease: Improving Global Outcomes, *NS* not significant

In Table 2, we assess if indirect methods were different among patients with and without MS. The patients with MS had lower eGDR, eIS and lnGDR values, and higher WHtR, VAI and TG/HDL-c values in comparison with patients without MS. Accordingly, a higher proportion of patients with MS had an eGDR < 7.32 mg/kg/min (60% vs. 9% in patients without MS, *p* < 0.001) and a WHtR > 0.52 (89% vs. 55% in patients without MS, *p* < 0.001). We also observed that eGDR and lnGDR were lower and TG/HDL-c was higher when comparing groups by sex. Despite eIS was lower in patients with MS, this result was statistically significant only in the female patients.

**Table 2:** Indirect markers of IR in patients with T1D with or without MS

	Total population	Without MS (*n* = 75)	With MS (*n* = 37)	*p*^a^
eGDR, mg/kg/min	8.39 (6.53–9.68)	8.93 (8.03–9.94)	5.49 (4.37–6.80)	< 0.001
Female	8.47 (6.79–9.92)	9.68 (8.35–10.15)	6.32 (5.1–6.99)	< 0.001
Male	7.78 (5.47–8.79)	8.69 (7.37–9.2)	4.41 (3.91–6.48)	< 0.001
WHtR	0.52 ± 0.06	0.50 ± 0.05	0.55 ± 0.05	0.001
eIS	3.27 (2.30–4.31)	3.51 (2.68–4.68)	2.89 (1.54–3.54)	0.007
Female	3.31 (2.42–4.19)	3.68 (2.72–4.33)	2.96 (1.27–3.54)	0.009
Male	3.14 (2.24–5.1)	3.40 (2.28–5.27)	2.85 (1.78–4.73)	NS
lnGDR, mg/kg/min	1.86 ± 0.27	1.95 ± 0.21	1.69 ± 0.27	< 0.001
Female	1.87 ± 0.26	1.96 ± 0.22	1.69 ± 0.27	0.001
Male	1.83 ± 0.27	1.93 ± 0.22	1.67 ± 0.29	0.016
TG/HDL-c	2.15 (1.38–3.65)	1.77 (1.18–2.75)	3.78 (2.63–5.73)	< 0.001
Female	1.99 (1.35–3.66)	1.51 (1.10–2.15)	3.99 (2.76–5.78)	< 0.001
Male	2.53 (1.60–3.70)	2.08 (1.32–3.21)	3.78 (2.53–5.22)	0.005
VAI	1.76 (1.13–2.99)	1.39 (0.97–1.92)	3.4 (1.92–5.70)	< 0.001

Data are presented as median (IQR) or mean ± SD. ^a^Statistically different among patients with or without MS with Student t test or Mann Whitney U according to their distribution. *eGDR* estimated glucose disposal rate, *WHtR* waist-to-height ratio, *eIS* estimated insulin sensitivity, *lnGDR* natural logarithm of glucose disposal rate, *TG/HDL-c* ratio triglycerides/HDL cholesterol, *VAI* visceral adiposity index

We performed ROC curves for eGDR, eIS, lnGDR, TG/HDL-c and VAI to determine the best cut-off points to detect MS. Table 3 depicts the best cut-off values, sensitivity, specificity, likelihood ratio positive (LR+) and likelihood ratio negative (LR-) values for each index. We corroborated that a cut-off point of < 7.32 mg/kg/min had the best AUC for detection of MS, followed by VAI, and TG/HDL-c. The lnGDR index in male and the TG/HDL-c index in female had the best cut-off points for MS detection.

**Table 3:** Best cut-off points for detection of MS in patients with T1D

Index	AUC (IC95%)	Cut-off value	Sensitivity	Specificity	LR+	LR-
eGDR, mg/kg/min	0.93 (0.87–0.98)	< 7.32	85%	84%	5.31	0.17
WHtR	0.70 (0.68–0.89)	> 0.52	77%	70%	2.58	0.33
eIS	0.68 (0.56–0.81)	< 2.92	70%	54%	1.52	0.55
Female	0.72 (0.57–0.86)	< 3.10	69%	66%	2.02	0.46
Male	0.63 (0.40–0.87)	< 2.92	72%	63%	1.94	0.44
lnGDR, mg/kg/min	0.77 (0.65–0.88)	< 1.8	82%	73%	3.03	0.24
Female	0.77 (0.64–0.91)	< 1.81	80%	69%	2.58	0.28
Male	0.80 (0.64–0.97)	< 1.77	88%	80%	4.4	0.15
TG/HDL-c	0.83 (0.76–0.91)	> 2.0	86%	64%	2.39	0.21
Female	0.86 (0.77–0.95)	> 2.2	81%	78%	3.68	0.24
Male	0.78 (0.63–0.93)	> 2.5	82%	62%	2.15	0.29
VAI	0.86 (0.78–0.94)	> 1.84	82%	71%	2.84	0.25

*eGDR* estimated glucose disposal rate, *WHtR* waist-to-height ratio, *eIS* estimated insulin sensitivity, *lnGDR* natural logarithm of glucose disposal rate, *TG/HDL-c* ratio triglycerides/HDL cholesterol, *VAI* visceral adiposity index

## Discussion

Using the Joint Statement criteria, 33% of patients with T1D in our clinic have MS. This prevalence is similar to the reported in the Metascreen Study in Italy (34%) using the AHA/NHLBI criteria [21], with the observed in a Spanish population (32%) using the National Cholesterol Education Program: Adult Treatment Panel III (NCEP: ATPIII) criteria [9] and with the reported in an American population in the DCCT/EDIC (Diabetes Control and Complications Trial/Epidemiology of Diabetes Interventions and Complications) study (36%) using IDF criteria [22]. We found that the most prevalent comorbidity in the patients with “double diabetes” was hypertension, even though it is a young population (mean age 35 years). The second most common comorbidity was central obesity, followed by low HDL-c levels, both of which are highly prevalent in the general Mexican population [23]. As expected, those patients also have a lower eGDR, eIS and lnGDR; and higher TG/HDL-c and VAI levels, suggesting higher IR. Among these methods, we observed that the most sensitive to detect MS in patients with T1D independently of gender, is eGDR. Additionally, in the female population the TG/HDL-c and in male population the lnGDR are also useful for this purpose.

The eGDR has been extensively validated in diverse populations [24] including in the DCCT/EDIC [22] and the EURODIAB cohorts [25]. Chillaron et al. reported that the number of MS traits inversely correlated with eGDR levels, with a Pearson correlation coefficient of − 0.793 (*p* < 0.001) [9]. In that study, a lower eGDR was associated with coronary artery disease and retinopathy in patients with T1D [9]. In this study, we corroborated that an eGDR level below 7.32 mg/kg/min had the highest sensitivity (85%) and specificity (84%) for MS detection, with the highest AUC in the ROC curve (AUROC 93%). In a Spanish cohort [9], an eGDR below 8.77 mg/kg/min had a similar specificity as our results (85%), but higher sensitivity (100%). Meanwhile, Tam et al. in an American cohort found that a cut-off point of 5.6 mg/kg/min had a lower sensitivity (75%) and specificity (71%) with an AUROC of 80% for defining IR [26]. As observed, the specific cut-off point of eGDR for IR detection and its utility vary depending the ethnicity and the statistical method used for its quantification, and is usually reported among 5 to 9 mg/kg/min [27].

In our study, the second most sensitive and specific method for detecting IR in total population was the lnGDR. This method was recently proposed by Zheng et al. and was calculated through a stepwise linear regression analysis using demographic and metabolic parameters. Its final formula incorporates HbA1c, DBP and WHR, who explain over 60% of the variance of GDR (adjusted R^2^ of 0.61, *p* < 0.01) [14]. For this index, we observed that a cut-off point of < 1.8 mg/kg/min had a high AUROC for MS detection (77%), especially in the male population in whom a cut-off point < 1.77 mg/kg/min had an AUROC of 80%. To our knowledge, this is the first study that evaluates this model in population other than Chinese. More studies are required to verify if this cut-off point has variations depending on ethnicity and its association with micro and macrovascular complications in patients with T1D.

We also assessed the TG/HDL-c and VAI indexes, whose main component are the lipids parameters [15]. Their utility in the detection of macrovascular complications has been studied in subjects with and without obesity [28, 29]. We found that a TG/HDL-c > 2.2 had the best sensitivity for MS in female population meanwhile a cut-off point of > 2.0 was also sensitive but with lower specificity for total population. As VAI formula has been adjusted for gender, we did not divide it in groups. We observed that a VAI > 1.84 had the same sensitivity as the lnGDR, but with lower specificity. In Poland, Uruska et al. studied 88 patients with T1D, with a mean age of 34 years and a median duration disease of 8 years (7–13 years). Using the HEC, IR was defined as GDR < 4 mg/kg/min and it was observed in 37.5% of the patients. Patients with IR had a higher TG/HDL-c [1.6 (1.0–1.3) vs. 1.05 (0.62–1.53), *p* = 0.001] and VAI [2.61 (1.31–4.25) vs 1.56 (0.96–2.25), *p* = 0.002], in comparison with patients without IR [15]. In that study the sensitivity and specificity for IR or MS detection was not provided, and it is the only one conducted in adult patients with T1D.

Regarding WHtR, in this study we corroborated that a cut-off point of 0.52 had a good sensitivity and specificity. This was previously demonstrated in our population [12], where it proved to have a better diagnostic utility in comparison with the waist circumference. This method could be particularly useful in populations were the average height varies by region.

Finally, we evaluated the utility of the eIS equation. This index was developed using clinical data from 36 adults with T1D recruited from the Coronary Artery Calcification in Type 1 Diabetes (CACTI) study, with a mean age of 45 ± 8 years and a mean duration of diabetes of 22.6 ± 7.8 years [30]. The original formula included WC, daily insulin dose (U/kg/day), adiponectin, TG and DBP. However, considering that patients are not always able to fast for their clinical visits a non-fasting model was created (eIS-nf) that include WC and daily insulin dose. Furthermore, an additional model was developed excluding adiponectin (eIS-eXA), because in some clinical scenarios (as in ours) it is not routinely measured. Once developed and validated, the eIS and its variants were tested in independent adult and adolescent populations and significatively correlated with clamp-measured insulin sensitivity, even with better correlation when compared to eGDR [13]. Bjornstad et al. further observed that greater eIS at baseline predicted lower odds of developing albuminuria, diabetic retinopathy proliferative diabetic retinopathy and progression of coronary artery calcium score [31]. A specific cut-off point for detecting IR in those studies has not been provided, but in our study the cut-off points proposed for total population or divided by gender had the lower sensitivity and specificity when compared with the other methods. Nevertheless, for this study we selected eIS-eXA. Assessing cut-off point for eIS and eIS-nf is pending.

The mechanisms involved in the development of IR in patients with T1D remain unknown [32]. Some studies have proposed that obesity due non-healthy lifestyle and over-insulinization in addition to the genetic background are its main cause [32]. Meanwhile others have reported that IR is present in patients with T1D even in the absence of obesity [6]. Indeed, it seems that patients with T1D have a decreased insulin sensitivity in liver tissue (defined in the HEC as a decreased insulin-stimulated suppression of endogenous glucose production), and an increased IR in muscle (observed in the HEC as a lower whole-body glucose disposal even in the presence of lower intrahepatic fat content) and in adipose tissue (observed due impaired insulin-mediated suppression of lipolysis with subsequent increase of free fatty acids and glycerol levels) [6, 32], in comparison with healthy controls. On the other hand, it has been proposed that IR is related with glucometabolic control, diabetes duration (more than 10 years) and ethnicity [33, 34]. However, those variables were not different among patients with T1D and “double diabetes” in our study.

The limitations of some of the reported methods should be taken into account. As analyzed by Gingras et al. [4], eGDR formula incorporates hypertension, whose presence could be overestimated due some patients use antihypertensive medications for cardiac or renal prevention. Furthermore, it also incorporates the waist-to-hip ratio, a measurement that varies depending of ethnicity. Regarding limitations of lnGDR and TG/HDL-c, the first also incorporates WHR; meanwhile a possible limitation for the second is that TG levels could widely vary depending on glycemic control. We overcome those biases, assessing that the treatment with antihypertensives was solely prescribed due to hypertension. Additionally, we believe hyperglycemia did not affect our results because there were no differences in fasting glucose or HbA1c levels among patients with or without MS.

Finally, the main limitation of our study is the lack of comparison with the HEC. Nevertheless, all the selected methods have been previously compared and validated with the gold standard. Moreover, our purpose was to identify the most accurate non-invasive and easy-to-perform method that could be useful for detect patients with double diabetes in a clinical setting. Our results support the use of eGDR in Mexican patients with T1D. However, more studies with larger cohorts and different ethnic groups are required to assess the utility of the others indirect markers, especially of those recently described as the lnGDR and eIS.

## Conclusion

In this Mexican cohort of adult patients with T1D an eGDR below 7.32 mg/kg/min showed the highest sensitivity and specificity for detection of MS (double diabetes). In female patients the TG/HDL-c > 2.2 and in male patients the lnGDR < 1.77 mg/kg/min are also useful for this purpose.

## Data Availability

The data analyzed in the study are available from the corresponding author on reasonable request.

## Abbreviations

T1D: Type 1 diabetes
T2D: Type 2 diabetes
IR: Insulin resistance
MS: Metabolic syndrome
GDR: Glucose disposal rate
HEC: Hyperinsulinemic euglycemic clamp
HOMA: Homeostatic model of assessment
eGDR: estimated glucose disposal rate
WHtR: Waist-to-height ratio
eIS: estimated insulin sensitivity index
TG/HDL-c: Triglycerides/high-density lipoprotein cholesterol ratio
VAI: Visceral adiposity index
SGLT-2: Sodium glucose co-transporter type 2
AHA/NHLBI: American Heart Association/ National Heart Lung and Blood Institute
IDF: International Diabetes Federation
WC: Waist circumference
WHR: Waist-to-hip ratio
BMI: Body mass index
WHO: World Health Organization
TC: Total cholesterol
HbA1c: Glycated hemoglobin
LDL-c: Low-density lipoprotein cholesterol
lnGDR: Natural logarithm of glucose disposal rate
DBP: Diastolic blood pressure
SD: Standard deviation
KDIGO: Kidney Disease: Improving Global Outcomes
ROC: Receiver Operating Characteristic
AUC: Area under curve
NPH: Neutral protamine Hagedorn insulin
GFR: Glomerular filtration rate
LR+: Likelihood positive ratio
LR-: Likelihood negative ratio
NCEP: ATPIII: National Cholesterol Education Program: Adult Treatment Panel III
DCCT/EDIC: Diabetes Control and Complications Trial/Epidemiology of Diabetes Interventions and Complications
CACTI: Coronary Artery Calcification in Type 1 Diabetes study
eIS-nf: estimated insulin sensitivity non-fasting model
eIS-exA: estimated insulin sensitivity without adiponectin model
